# ﻿Three new species of *Retusigaster* Dangerfield, Austin & Whitfield, 1999 (Hymenoptera, Braconidae, Cardiochilinae) with an illustrated key to the New World species

**DOI:** 10.3897/zookeys.1092.80560

**Published:** 2022-04-04

**Authors:** Ilgoo Kang

**Affiliations:** 1 Department of Entomology, Louisiana State University Agricultural Center, 404 Life Sciences Building, Baton Rouge, LA, 70803 USA Louisiana State University Agricultural Center Baton Rouge United States of America

**Keywords:** *Gossypium* sp., parasitoid wasps, *Purshiamexicana*, taxonomy

## Abstract

*Retusigaster* Dangerfield, Austin & Whitfield, 1999 is a genus of the subfamily Cardiochilinae Ashmead, 1900 and exhibits high species richness in the New World. Eight species of the genus were recorded before this work: five species from the Nearctic region, two species from the Neotropical region, and one species from the Palearctic region. In this article, three new species of New World *Retusigaster* are described based on morphological characters: *R.pulawskii***sp. nov.**; *R.purshi***sp. nov.**; *R.vanduzeei***sp. nov.** In addition, potential food sources of the members of *R.arugosus* (Mao, 1949) and *R.purshi***sp. nov.** are reported, and an illustrated key to the New World species of *Retusigaster* is provided. The number of species of *Retusigaster* in the New World is increased from seven to ten.

## ﻿Introduction

*Retusigaster* Dangerfield, Austin & Whitfield, 1999 is a genus of Cardiochilinae Ashmead, 1900 (Ashmead 1900) with eight valid species (Yu et al. 2016). Seven species were recorded from the New World: *R.arugosus* (Mao, 1949), *R.albopilosus* Mercado, 2003, *R.brevitarsus* (Mao, 1949), *R.dignus* (Mao, 1949), *R.noguerai* Mercado, 2003, *R.pullus* (Mao, 1949) and *R.rubidus* (Mao, 1949). One species, *R.eremita* (Kokujev, 1904) was recorded from the Palearctic region. Genus-level phylogenetic analyses, based on morphological data, were conducted by Dangerfield et al. (1999) and Mercado and Wharton (2003) and validated the genus. In the phylogeny of Dangerfield et al. (1999), which included representatives of all the cardiochiline genera in the world, *Retusigaster* was resolved as a monophyletic group. In the phylogeny of Mercado and Wharton (2003), *Retusigaster* species groups, which were defined by the authors based on the degree of thickening of the apex of hind tibia were clustered with the members of *Toxoneuron* Say, 1836 (Say 1836) but made *Toxoneuron* paraphyletic. Regarding the result of their analysis, Mercado and Wharton (2003) described that “Nevertheless, *Retusigaster*, as defined by its type species *rubidus*, is readily identifiable and is accepted here as a monophyletic group regardless of the rank eventually accorded.” In the current project, I follow the definition of Dangerfield et al. (1999) and describe three new species collected in the New World. Potential food sources of *R.arugosus* and *R.purshi* sp. nov. are reported, and an illustrated key to the New World species is included. In addition, the species groups defined by Mercado and Wharton (2003) with their diagnostic characters are re-evaluated and discussed, and the placement of *R.eremita* is discussed.

## ﻿Materials and methods

### ﻿Specimen information

The specimens for this work were borrowed from the California Academy of Sciences (**CAS**; San Francisco, CA, USA), Hymenoptera Institute (**HIC**; Redlands, California, USA), Museum of Comparative Zoology (**MCZ**; Cambridge, Massachusetts, USA), and Texas A&M University Insect Collection (**TAMU**; College Station, Texas, USA). Types of the new species will be deposited in CAS and the National Museum of Natural History (**NMNH**; the Smithsonian Institution, Washington D.C., USA).

### ﻿Morphological analyses

Specimens were examined using a Leica MZ75 stereomicroscope. The morphological terminology follows Dangerfield et al. (1999) and Sharkey and Wharton (1997). The terms used in this work can be found as synonyms on the website of Hymenoptera Anatomy and Consortium (2022). Terms for surface sculpture are based on Harris (1979). The following acronyms are used for morphological terms: POL: distance between posterior ocelli, T2: second metasomal tergum, and T3: third metasomal tergum. Using a Visionary Digital BK Plus imaging system (Dun, Inc.) with a Canon EOS 5DS DSLR, images were captured. Image stacking was performed via Zerene Stacker v.1.04 (Zerene Systems LLC.). Images were edited using Adobe Photoshop CS 6 and Photoshop CC 2022 v. 23.0 (Adobe Systems, Inc), and final image plates were produced using the same Adobe software. Body parts were measured using the same Adobe software mentioned. Numbers in parentheses in species descriptions indicate 0.01× the actual size of each body character. The unit of length is mm.

## ﻿Results

### ﻿Taxonomy

#### ﻿ Retusigaster

Taxon classificationAnimaliaHymenopteraBraconidae

Dangerfield, Austin & Whitfield, 1999

DF5DF810-F4FA-5AB6-BD87-D06ED4206DD2

##### Type species.

*Cardiochilesrubidus* Mao, 1949

##### Diagnosis.

Dangerfield et al. (1999) and Mercado and Wharton (2003) provided detailed diagnostic characters. *Retusigaster* can be easily distinguished from other cardiochiline genera by the combination of the following characters: eye seemingly bare (Figs 2C, 3C, 5C); clypeal tubercle absent (Figs 2C, 3C, 5C); mouthparts short (Figs 2C, 3C, 5C); scutellum apically with carinate margin (Key image 2); hind tibia without apical cuplike projection (Figs 2A, 3A, 5A); ovipositor and ovipositor sheath short (Figs 2A, 3A, 5A); hypopygium entirely sclerotized and ventro-apically blunt (Figs 2A, 3A, 5A).

##### Distribution.

Nearctic region (Canada, USA, Mexico), Neotropical region (Jamaica and Mexico), Palearctic region (Kazakhstan, Mongolia, Turkey, Turkmenistan).

##### Biology.

Potential food sources of two species of *Retusigaster* are found. A member of *R.arugosus* was collected on cotton (*Gossypium* sp.; Malvaceae) in Texas, and a specimen of *R.purshi* sp. nov. was collected on Mexican cliffrose (*Purshiamexicana* (D. Don) S. L. Welsh; Rosaceae) in Nevada.

### ﻿Key to species of *Retusigaster* of the New World

**Table:** 

1	Metasoma mostly or entirely pale	**2**
–	Metasoma mostly or entirely dark	**5**
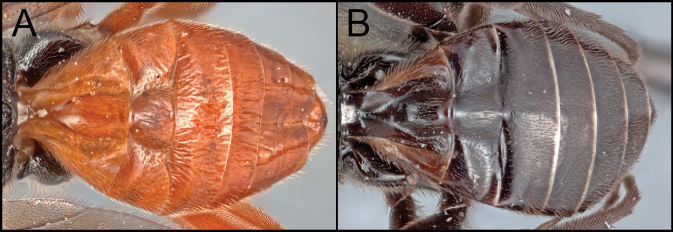		
2	Mesoscutum mostly or entirely pale	**3**
–	Mesoscutum mostly or entirely dark	**4**
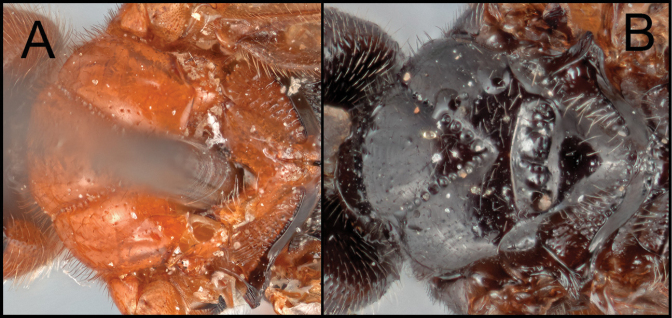		
3	Notauli smooth	***R.brevitarsus***
–	Notauli crenulate	***R.rubidus***
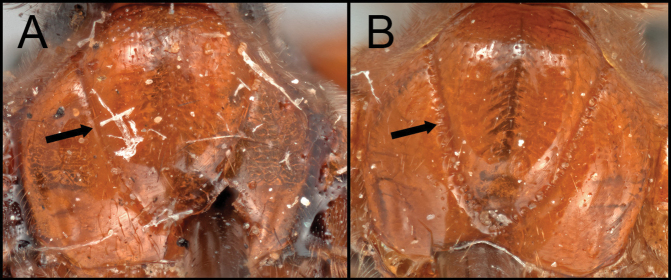		
4	Fore wing apically infuscate	***R.arugosus***
–	Fore wing entirely infuscate	***R.pullus***
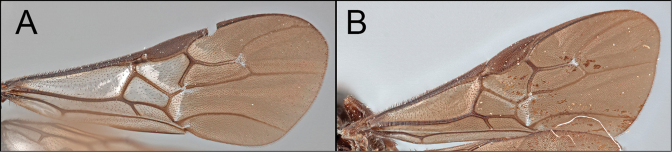		
5	Fore wing apically infuscate	**6**
–	Fore wing entirely infuscate	**9**
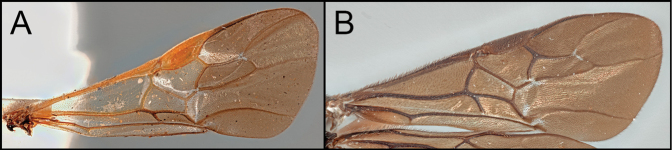		
6	Stigma entirely pale	***R.dignus***
–	Stigma entirely dark	**7**
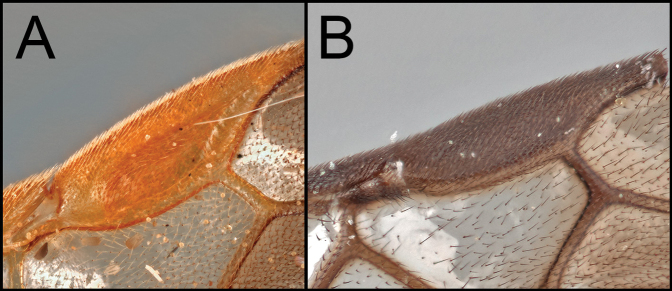		
7	Fore tibia entirely dark	***R.purshi* sp. nov.**
–	Fore tibia entirely pale	**8**
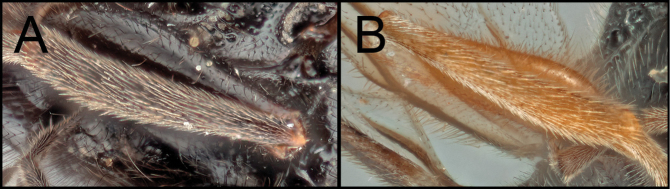		
8	Metafemur entirely pale	***R.pulawskii* sp. nov.**
–	Metafemur entirely dark	***R.albopilosus***
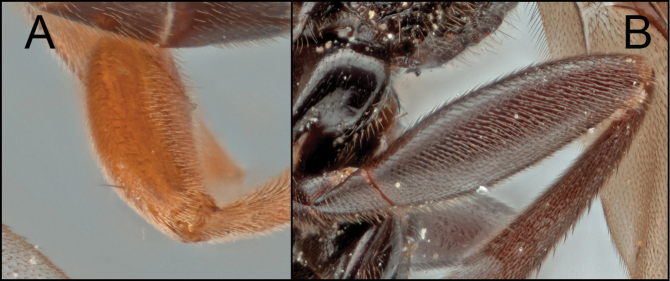		
9	Fore tibia entirely black	***R.vanduzeei* sp. nov.**
–	Fore tibia entirely pale	***R.noguerai***
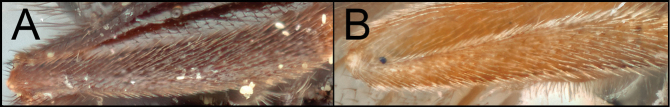		

### ﻿Species descriptions

#### ﻿ Retusigaster albopilosus

Taxon classificationAnimaliaHymenopteraBraconidae

Mercado, 2003

C5E96918-3AD6-50EF-A2C7-F34AE05BA254

##### Material examined.

***Paratypes*** Mexico • 2 ♀; Xmatkuil, Mérida, Yucatán; 25–28.v.1996; Wharton & León; Malaise Trap. Deposited in TAMU.

##### Diagnosis.

Members of *Retusigasteralbopilosus* can be recognized by the combination of the following characters: body 3.5–5.5 mm; fore wing entirely infuscate with dark stigma; fore tibia pale; mid and hind femur entirely dark; T2 entirely dark.

##### Description.

See Mercado and Wharton (2003).

**Male.** See Mercado and Wharton (2003).

##### Biology

. Unknown.

##### Distribution.

Neotropical region (Mexico).

#### ﻿ Retusigaster arugosus

Taxon classificationAnimaliaHymenopteraBraconidae

(Mao, 1949)

B1E7AEEF-8B6D-5D66-A019-9C253C3CE52D

##### Material examined.

***Non-type specimens*** USA: 1♀; Lexington, Massachusetts; 8.ix.1963; H. E. Evans. Deposited in MCZ. 1♀; only collected location was labelled (Chicago). Deposited in MCZ. 1♀; only collected month was labelled (July). Identified as *Cardiochilesabdominalis* Cresson by a previous examiner. Deposited in MCZ. 1♀; near Rio Frio, Garner State Park, Uvalde Co.; 21.vii.1986; 1400’; Wooley & Zolnerowich. Deposited in TAMU. 1♀; Brazos County, Texas; 25.vi.1937; J. E. Gillaspy. Deposited in TAMU.

##### Diagnosis.

*Retusigasterarugosus* is nearly identical to *R.pullus*. The members of both species possess dark head and metasoma with pale metasoma. As Mao (1949) mentioned, *R.arugosus* is distinguished from *R.pullus* by having basally hyaline and apically infuscate wings (Key image 4A). Body ~ 5.5 mm.

##### Description.

See Mao (1949).

**Male.** Unknown.

##### Biology

**(potential food source).** Cotton (*Gossypium* sp.; Malvaceae; recorded on the label of one specimen collected in Brazos County, Texas).

##### Distribution.

Nearctic region (Canada, USA).

#### ﻿ Retusigaster brevitarsis

Taxon classificationAnimaliaHymenopteraBraconidae

(Mao, 1949)

226B65F9-5ECC-5F6E-B6A9-E913AA92B2A4

##### Material examined.

***Non-type specimens*** USA: 1♀; Saugus, Los Angeles, California; 18.viii.1917; J. Bequaers. Deposited in MCZ. 1♀; Warren, San Diego, California; 13.viii.1917; J. Bequaers. Deposited in MCZ.

##### Diagnosis.

Members of *Retusigasterbrevitarsis* are most similar to *R.rubidus*. *Retusigasterbrevitarsis* can be distinguished from other members of the genus by the following characters: body length (~ 7.0mm); notauli smooth (Key image 3A); mesoscutum mostly orange pale; forewing entirely infuscate with dark (Key image 5B); metasoma mostly orange pale.

##### Description.

See Mao (1949).

**Male.** Unknown.

##### Biology.

Unknown.

##### Distribution.

Nearctic region (USA).

#### ﻿ Retusigaster dignus

Taxon classificationAnimaliaHymenopteraBraconidae

(Mao, 1949)

0FB82418-F7EE-59DD-995D-95EB78E28C15

Fig. 1

##### Material examined.

***Non-type specimen*** USA: 1♀; Pearsall, Texas. 30.ix.1936. Deposited in TAMU.

##### Diagnosis.

**Figure 1.**
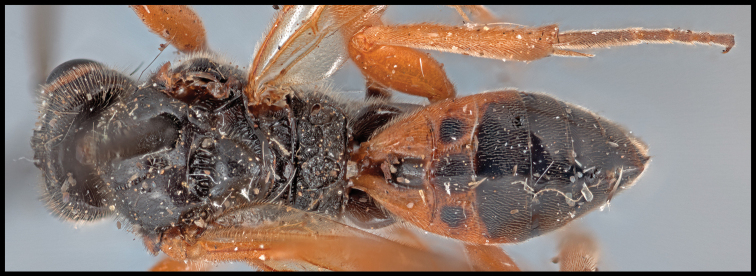
Dorsal habitus of *Retusigasterdignus*.

*Retusigasterdignus* can be distinguished from other members of *Retusigaster* by having longer body length (~ 7.5mm); fore wing apically infuscate with pale stigma (Key Image 5A); basal spur on hind tibia 0.67 × longer than length of basitarsus; T1 pale; T2 mostly pale, medially and submedially dark (Fig. 1).

##### Description.

See Mao (1949).

**Male.** Unknown.

##### Biology.

Unknown.

##### Distribution.

Nearctic region (USA).

#### ﻿ Retusigaster noguerai

Taxon classificationAnimaliaHymenopteraBraconidae

Mercado, 2003

430F15B1-3447-5B9C-AEEE-9ED3E55BB7B2

##### Material examined.

***Paratype*** Mexico: 1♀; Estación de Biología Chamela, Jalisco, Mexico; 3–9.vii.1993; Wharton & Sharkey. ***Non-type specimen*** Mexico • 1♀; same as previous except for collecting date and collector. 8.vii.1994; I. Mercado. Deposited in TAMU.

##### Diagnosis.

*Retusigasternoguerai* is similar to *R.vanduzeei* sp. nov. *R.noguerai* can be distinguished from other members of the genus by the combination of the following characters: body 4.5–6.0 mm; fore wing entirely infuscate with dark stigma; fore femur and tarsus pale; metasoma mostly dark; T2 1.0–1.3 × longer than its posterior width.

##### Description.

See Mercado and Wharton (2003).

**Male.** See Mercado and Wharton (2003).

##### Host.

Unknown.

##### Distribution.

Neotropical region (Mexico).

#### ﻿ Retusigaster pulawskii

Taxon classificationAnimaliaHymenopteraBraconidae

Kang sp. nov.

EAAB0BD2-200B-5564-AF0C-2A526F9D74AC

http://zoobank.org/B6B59B1C-6EFE-4793-BA8B-0D5DB4A67502

Fig. 2A–G

##### Material examined.

***Holotype*** Jamaica: ♀; Port Henderson, Catherine Parish; 16.xi.1986; W. J. Pulawski. Holotype will be deposited in CAS.

##### Diagnosis.

**Figure 2.**
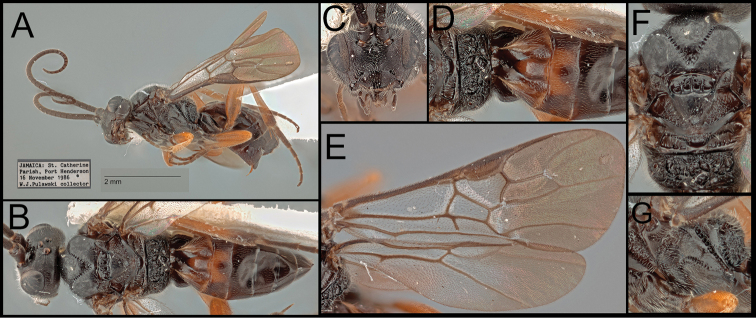
*Retusigasterpulawskii* sp. nov. **A** lateral habitus **B** dorsal habitus **C** anterior head **D** dorsal propodeum to T3 **E** wings **F** dorsal mesosoma **G** mesopleuron and metapleuron.

*Retusigasterpulawskii* sp. nov. is most similar to *R.albopilosus*. The following characters can distinguish the new species from other species of *Retusigaster*: precoxal sulcus not reaching posterior margin of mesopleuron (Fig. 2G); fore tibia entirely pale (Fig. 2A); fore wing apically infuscate (Fig. 2E); transverse carina of propodeum reaching lateral margin (Figs 2D, 2F); stigma entirely dark (Fig. 2E); hind femur entirely pale (Fig. 2A); metasoma mostly dark (Fig. 2B); T1 laterally orange (Fig. 2D); Y-shaped suture of T1entirely smooth (Fig. 2D); T2 medially orange (Fig. 2D).

##### Description.

Body ~ 4.69 mm. ***Head***: Antenna 37-segmented. Face width ~ 1.28 × longer than its height (73:57). Interantennal space with median carina. Width of anterior ocellus ~ 0.92 × longer than POL (11:12). Eye seemingly without interommatidial setae; median width of eye ~ 0.97 × longer than the median width of gena in lateral view (31:32). Gena extended ventroposteriorly into weak prominence. Clypeus ~ 2.25 × longer than its height (54:24); clypeal tubercles absent. Mandible bidentate. Maxillary palpus five-segmented. Labial palpus four-segmented. Galea short. Glossa short. Occipital carina absent. ***Mesosoma***: Notauli entirely crenulate, strongly crenulate posteriorly. Scutellar sulcus ~ 0.44 × longer than width (19:43), with three carinae. Postscutellar depression finely crenulate. Pronotum dorsally crenulate and posteriorly rugulose. Mesopleulon mostly smooth, posterior margin strongly crenulate; precoxal sulcus crenulate not reaching posterior margin; epicnemial carina absent; episternal scrobe present. Metapleuron anteriorly smooth and posteriorly rugulose. Propodeum strongly rugulose, ~ 0.36 × longer than its median width (36:100); propodeal areola heart-shaped, ~ 1.17 × longer than its maximum width (27:23); transverse carina reaching lateral margin. ***Legs***: Basal spur on fore tibia ~ 0.58 × longer than length of basitarsus (19:33). Basal spur on mid tibia ~ 0.63 × longer than length of basitarsus (30:48). Hind tibia without apical cup-like projection; basal spur on hind tibia ~ 0.64 × longer than length of basitarsus (47:74); claws pectinate. ***Wings***: Fore wing ~ 4.14 mm; second submarginal cell trapezoid, ~ 3.20 × longer than height (80:25); 1r absent; 3r absent; RS evenly curved; pterostigma ~ 2.89 × longer than wide medially (81:28). Hind wing ~ 3.43 mm; 2r-m absent; 2–1A basally present. ***Metasoma***: T1 ~ 1.32 × longer than its posterior width (66:50), anteriorly with lateral carina; Y-shaped suture of T1entirely smooth. T2 ~ 0.34 × longer than its posterior width (36:105), ~ 0.95 × longer than T3 (36:38). T3 ~ 0.36 × longer than its posterior width (38:106). Hypopygium without median longitudinal fold. Protruded ovipositor sheath ~ 0.27 × longer than length of hind basitarsus (20:74), apically with short setae. ***Color***: Body mostly dark brown. Fore wing apically infuscate; stigma entirely dark. The following areas orange: fore tibia; all femora, basal mid and hind tibiae; medial and lateral T1; medial T2.

##### Etymology.

Named in honor of Dr Wojciech Jerzy Pulawski, Curator of Entomology, Emeritus, at CAS, the person who collected the specimen from Jamaica.

##### Biology.

Unknown.

##### Distribution.

*Retusigasterpulawskii* sp. nov. is known from a single female specimen collected in Jamaica.

#### ﻿ Retusigaster pullus

Taxon classificationAnimaliaHymenopteraBraconidae

(Mao, 1949)

C628C157-5C23-51A4-B0E4-33BE24FADCD0

##### Material examined.

***Non-type specimens*** USA • 1♀; three miles east of Presidio, Texas; 1–3.v.1963; H. E. Evans. Deposited in MCZ. 1♀; Randall County, Texas; Bushland; 26.vii.−7.viii.1983; T. J. Kring; Malaise trap. Deposited in TAMU.

##### Diagnosis.

By having dark head and mesosoma with pale metasoma, members of *R.pullus* and *R.arugosus* can be distinguished from the other members of *Retusigaster*. The members of *R.pullus* are distinguished from the members of *R.arugosus* by having entirely infuscate wings (Key image 4B).

##### Description.

See Mao (1949).

**Male.** Unknown.

##### Biology.

Unknown.

##### Distribution.

Nearctic region (USA).

#### ﻿ Retusigaster purshi

Taxon classificationAnimaliaHymenopteraBraconidae

Kang sp. nov.

80E55D77-1C7E-5607-A735-4C42C470D859

http://zoobank.org/F46EE693-08F0-45D5-ABEF-0E30F533153B

Fig. 3A–E

##### Material examined.

***Holotype*** USA: ♀; 36°16.15'N, 115°33.29'W; Telephone Canyon, Clark County, Nevada, USA; 16.vi.1998; K. Keen & M. Andres; Collected on *PurshiaMexicana*. Holotype will be deposited in NMNH.

##### Diagnosis.

**Figure 3.**
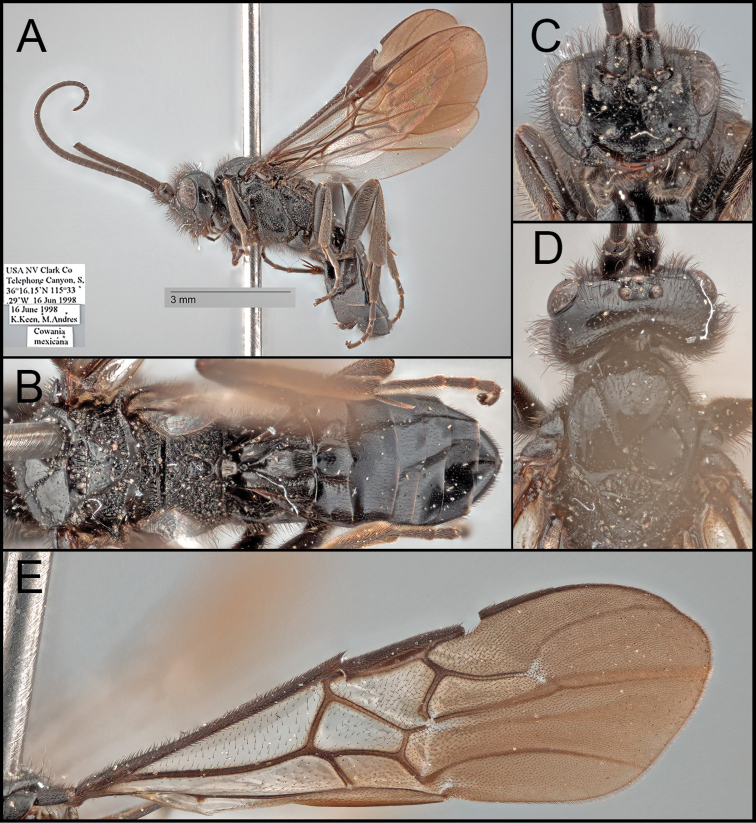
*Retusigasterpurshi* sp. nov. **A** lateral habitus **B** dorsal habitus **C** anterior head **D** dorsal mesosoma **E** forewing.

*Retusigasterpurshi* sp. nov. is most similar to *R.vanduzeei* sp. nov. The following characters can distinguish *R.purshi* sp. nov. from other species of *Retusigaster*: body ~ 7.0 mm, mostly black except for medial mandible (reddish brown) and ovipositor (Fig. 3A, C); precoxal sulcus crenulate reaching posterior margin (Fig. 3A); propodeal areola pentagonal (Fig. 3B); fore wing apically infuscate with dark stigma (Fig. 3A); fore tibia entirely dark; Y-shaped suture posteriorly crenulate (Fig. 3B).

##### Description.

Body ~ 7.06 mm. ***Head***: Head entirely with long setae. Antenna 44-segmented. Face width ~ 1.56 × longer than its height (128:82). Width of anterior ocellus ~ 0.70 × longer than POL (16:23). Eye seemingly without interommatidial setae; median width of eye about ~ 0.90 × longer than the median width of gena in lateral view (47:52). Gena extended ventro-posteriorly into moderate prominence. Clypeus ~ 2.46 × longer than its height (96:39), with punctures; clypeal tubercles absent. Mandible bidentate. Maxillary palpus five-segmented. Labial palpus four-segmented. Galea short. Glossa short. Occipital carina absent. ***Mesosoma***: Notauli entirely evenly crenulate. Scutellar sulcus ~ 0.45 × longer than width (28:62), with seven carinae, posteriorly rugulose. Postscutellar depression dorsally rugulose and ventrally crenulate. Pronotum dorsally crenulate and posteriorly rugulose. Mesopleuron dorsally with punctures and ventrally crenulate and rugulose, posterior margin strongly crenulate; precoxal sulcus crenulate reaching posterior margin; epicnemial carina absent; episternal scrobe present. Metapleuron anteriorly smooth and posteriorly rugulose. Propodeum strongly rugulose, ~ 0.38 × longer than its median width (61:162); propodeal areola pentagonal, ~ 1.45 × longer than its maximum width (48:33); transverse carina reaching lateral margin. ***Legs***: Basal spur on mid tibia ~ 0.64 × longer than length of basitarsus (40:63). Hind tibia without apical cup-like projection; basal spur on hind tibia ~ 0.61 × longer than length of basitarsus (57:93); claws pectinate. ***Wings***: Fore wing ~ 6.59 mm; second submarginal cell trapezoid, ~ 3.02 × longer than height (124:41); 1r present as basal stump; 3r absent; RS evenly curved; pterostigma about 3.00 × longer than wide medially (105:35). Hind wing ~ 4.57 mm; 2r-m absent; 2–1A present reaching basal half. ***Metasoma***: T1 ~ 1.01 × longer than its posterior width (93:92), anteriorly with lateral carina; Y-shaped suture of T1 anteriorly smooth and posteriorly crenulate. T2 ~ 0.27 × longer than its posterior width (43:158), ~ 0.77 × longer than T3 (43:56). T3 ~ 0.34 × longer than its posterior width (56:164). Hypopygium without median longitudinal fold. Protruded ovipositor sheath ~ 0.29 × longer than length of hind basitarsus (27:93), apically with long setae. ***Color***: Body mostly black. Wings basally hyaline and apically infuscate. Pterostigma entirely dark brown. Mandible apically black. Apical tarsomeres pale.

##### Etymology.

Named in honor of Fredrick Traugott Pursh, a German American botanist. The genus of the potential food source was also named after him, *Purshia*.

##### Biology

**(potential food source).** Mexican Cliffrose (*Purshiamexicana* (D. Don) S. L. Welsh; Rosaceae)

##### Distribution.

**Figure 4.**
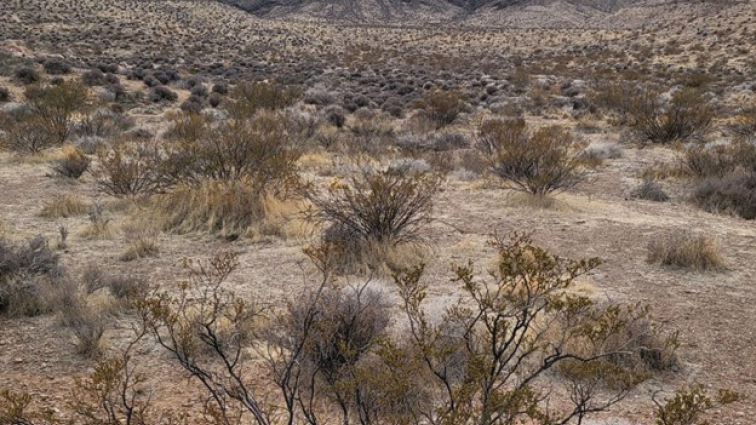
Habitat near the type locality of *Retusigasterpurshi* sp. nov. in Nevada, USA.

*Retusigasterpurshi* sp. nov. is known from one female specimen collected in Telephone Canyon, Clark County, Nevada, USA. (Fig. 4)

#### ﻿ Retusigaster rubidus

Taxon classificationAnimaliaHymenopteraBraconidae

(Mao, 1949)

F5EBE3D3-CF0B-54FE-A21A-8AFEA0A21FD8

##### Material examined.

***Non-type specimens*** Mexico • 3♀; seven miles east of San Luis Potosí; 3.vii.1987; 6225’; R. Wharton. USA • 1♀; same as previous except for collecting date and collector. 8.vii.1994; I. Mercado. Deposited in TAMU.

##### Diagnosis.

Members of *Retusigasterrubidus* are most similar to those of *R.brevitarsis*. *R.rubidus* can be distinguished from other members by the following characters: body ~ 7.5 mm. notauli crenulate (Key image 3B); mesoscutum mostly orange pale; fore wing with pale stigma (Key image 6A); metasoma mostly orange pale.

##### Description.

See Mercado and Wharton (2003).

**Male.** Unknown.

##### Host.

Unknown.

##### Distribution.

Nearctic region (USA and Mexico).

#### ﻿ Retusigaster vanduzeei

Taxon classificationAnimaliaHymenopteraBraconidae

Kang sp. nov.

10FD02B0-C2A1-5CEC-8011-26714A5E67B6

http://zoobank.org/6CF56EA2-C9D0-4CEA-B780-17D6D6613E66

Fig. 5A–H

##### Material examined.

***Holotype*** USA • ♀; Nixon, Washoe County, Nevada; 30.vi.1927; EP Van Duzee. Holotype will be deposited in CAS.

##### Diagnosis.

**Figure 5.**
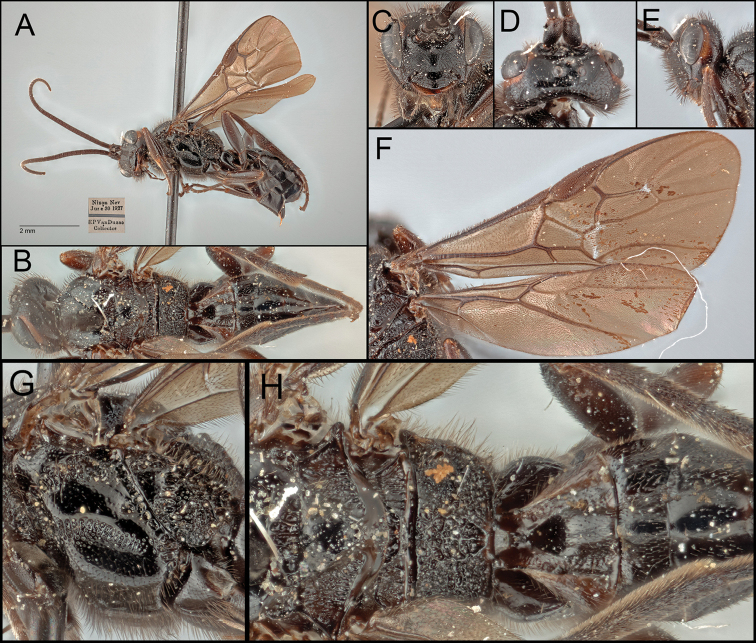
*Retusigastervanduzeei* sp. nov. **A** lateral habitus **B** dorsal habitus **C** anterior head **D** dorsal head **E** lateral head **F** wings **G** mesopleuron and metapleuron **H** scutellum to T3.

*Retusigastervanduzeei* sp. nov. is most similar to *R.noguerai* Mercado. Using the following characters, the members of *R.vanduzeei* sp. nov. can be distinguished from other members the genus: inner and outer orbits orange (Fig. 5A–E); fore wing entirely infuscate (Fig. 5F); precoxal sulcus crenulate nearly reaching posterior margin (Fig. 5G); propodeal areola oval (Fig. 5H); metasoma entirely dark (Fig. 5B); T1 antero-laterally crenulate and postero-laterally slightly rugulose; T2 ~ 0.27 × longer than its posterior width (Fig. 5B, H).

##### Description.

Body ~ 6.32 mm. ***Head***: Head entirely with long setae. Antenna 37-segmented. Face width ~ 1.50 × longer than its height (96:64). Width of anterior ocellus ~ 0.80 × longer than POL (16:20). Eyes seemingly without interommatidial setae; median width of eye about ~ 1.15 × longer than the median width of gena in lateral view (45:39). Gena extended ventro-posteriorly into moderate prominence. Clypeus ~ 2.67 × longer than its height (72:27), with punctures; clypeal tubercles absent. Mandible bidentate. Maxillary palpus five-segmented. Labial palpus four-segmented. Galea short. Glossa short. Occipital carina absent. ***Mesosoma***: Notauli entirely evenly crenulate. Scutellar sulcus ~ 0.30 × longer than width (22:74), with six carinae; lateral margins forming cup-like pit posteriorly. Postscutellar depression entirely rugulose. Pronotum mostly rugulose. Mesopleuron dorsally and ventrally with punctures, posterior margin strongly crenulate; precoxal sulcus crenulate nearly reaching posterior margin; epicnemial carina absent; episternal scrobe present. Metapleuron anteriorly smooth and posteriorly rugulose. Propodeum strongly rugulose, ~ 0.40 × longer than its median width (57:142); propodeal areola nearly oval, ~ 1.31 × longer than its maximum width (42:32); transverse carina absent. ***Legs***: Basal spur on fore tibia ~ 0.58 × longer than length of basitarsus (29:50). Basal spur on mid tibia ~ 0.64 × longer than length of basitarsus (39:61). Hind tibia without apical cup-like projection; basal spur on hind tibia ~ 0.61 × longer than length of basitarsus (55:90); claws pectinate. ***Wings***: Fore wing ~ 6.06 mm; second submarginal cell trapezoid, ~ 3.06 × longer than height (110:36); 1r absent; 3r absent; RS evenly curved; pterostigma about ~ 3.34 × longer than wide medially (117:35). Hind wing ~ 4.88 mm; 2r-m absent; 2–1A present reaching basal half. ***Metasoma***: T1 ~ 1.13 × longer than its posterior width (79:70), antero-laterally crenulate and postero-laterally slightly rugulose. T2 ~ 0.27 × longer than its posterior width (37:136), ~ 0.55 × longer than T3 (37:67). Hypopygium without median longitudinal fold. Protruded ovipositor sheath ~ 0.46 × longer than length of hind basitarsus (41:90), apically setaceous. **Color**: Body mostly black. Wings entirely infuscate. Pterostigma entirely dark brown. Antenna dark brown. Inner and outer orbits orange. Mandible medially reddish brown. First laterotergite brown.

##### Etymology.

Named in honor of Mr Edward P. Van Duzee, a former curator of CAS and fellow of Entomological Society of America (ESA), the person who collected the specimen.

##### Host.

Unknown.

##### Distribution.

*Retusigastervanduzeei* sp. nov. is known from Nixon, Washoe County, Nevada, USA.

## ﻿Discussion

**Figure 6.**
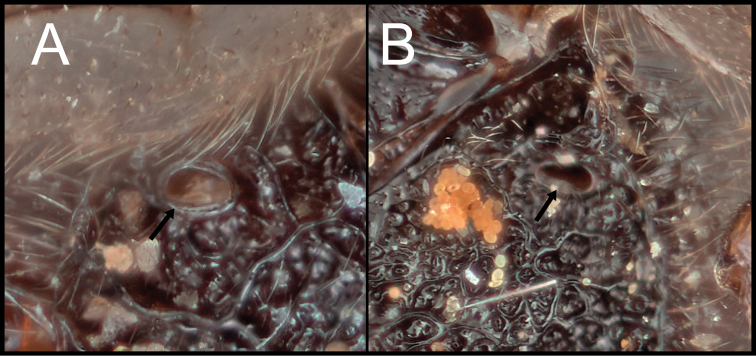
**A** propodeal spiracle of *R.arugosus***B** propodeal spiracle of *R.vanduzeei* sp. nov.

Mercado and Wharton (2003) separated the two species groups, *R.arugosus* and *R.rubidus*, by the degree of expansion of the apex of hind tibia and the shape and location of the propodeal spiracles as mentioned in the introduction section. I examined and compared the diagnostic characters of all the species of *Retusigaster* designated by Mercado and Wharton (2003). In my examination, I did not see a distinct difference in the hind tibial character between the two groups. The shape of the propodeal spiracles (Fig. 6) was somewhat useful to identify the species groups rather than the location of the propodeal spiracle, but still it was not easy to confidently distinguish two species groups based on the shape. Accordingly, the new species are placed neither in *R.arugosus* nor in *R.rubidus* due to the difficulties separating the two species groups based on the suggested diagnostic characters by Mercado and Wharton (2003). Further research based on molecular data will clarify the relationships among species and species groups of *Retusigaster* and *Toxoneuron*.

Regarding the placement of *R.eremita*, Mercado and Wharton (2003) were unsure of the placement because the species is only a member of *Retusigaster* recorded from the Palearctic region. I was also inquisitive about its generic placement and reviewed the species descriptions by Telenga (1955) and Tobias (1995) to reconfirm the placement of the species. Telenga (1955) wrote that the members of *R.eremita* possess much thickened tips of hind tibiae and simple claws, which have not been recorded in other species of *Retusigaster*. Both of the characters suggest placement in *Pseudcardiochilus* Hedwig, 1957 among the Old World cardiochilines. According to the description by Tobias (1995), the members of *R.eremita* have simple claws but the apices of their hind tibia are not as expanded as those of *Pseudcardiochilusacutus* (Tobias & Alexeev, 1977). However, because I did not examine type specimens of *R.eremita*, I do not change the generic placement of the species. Erdoğan (2015) reported the first record of *R.eremita* from Turkey, but the species may not be *R.eremita* because of its extremely different body coloration.

## Supplementary Material

XML Treatment for Retusigaster

XML Treatment for Retusigaster albopilosus

XML Treatment for Retusigaster arugosus

XML Treatment for Retusigaster brevitarsis

XML Treatment for Retusigaster dignus

XML Treatment for Retusigaster noguerai

XML Treatment for Retusigaster pulawskii

XML Treatment for Retusigaster pullus

XML Treatment for Retusigaster purshi

XML Treatment for Retusigaster rubidus

XML Treatment for Retusigaster vanduzeei
